# Performance of Multiple Metagenomics Pipelines in Understanding Microbial Diversity of a Low-Biomass Spacecraft Assembly Facility

**DOI:** 10.3389/fmicb.2021.685254

**Published:** 2021-09-28

**Authors:** Jason M. Wood, Nitin K. Singh, Lisa Guan, Arman Seuylemezian, James Nick Benardini, Kasthuri Venkateswaran

**Affiliations:** Biotechnology and Planetary Protection Group, Jet Propulsion Laboratory, California Institute of Technology, Pasadena, CA, United States

**Keywords:** planetary protection, metagenomics, Spacecraft Assembly Facility, microbial diversity, low-biomass

## Abstract

NASA planetary protection (PP) requires an assessment of the biological contamination of the potential microbial burden on spacecraft destined to explore planetary bodies that may harbor signs of life, like Mars and Europa. To help meet these goals, the performance of multiple metagenomic pipelines were compared and assessed for their ability to detect microbial diversity of a low-biomass clean room environment used to build spacecraft destined to these planetary bodies. Four vendors were chosen to implement their own metagenomic analysis pipeline on the shotgun sequences retrieved from environmental surfaces in the relevant environments at NASA’s Jet Propulsion Laboratory. None of the vendors showed the same microbial profile patterns when analyzing same raw dataset since each vendor used different pipelines, which begs the question of the validity of a single pipeline to be recommended for future NASA missions. All four vendors detected species of interest, including spore-forming and extremotolerant bacteria, that have the potential to hitch-hike on spacecraft and contaminate the planetary bodies explored. Some vendors demonstrated through functional analysis of the metagenomes that the molecular mechanisms for spore-formation and extremotolerance were represented in the data. However, relative abundances of these microorganisms varied drastically between vendor analyses, questioning the ability of these pipelines to quantify the number of PP-relevant microorganisms on a spacecraft surface. Metagenomics offers tantalizing access to the genetic and functional potential of a microbial community that may offer NASA a viable method for microbial burden assays for planetary protection purposes. However, future development of technologies such as streamlining the processing of shotgun metagenome sequence data, long read sequencing, and all-inclusive larger curated and annotated microbial genome databases will be required to validate and translate relative abundances into an actionable assessment of PP-related microbes of interest. Additionally, the future development of machine learning and artificial intelligence techniques could help enhance the quality of these metagenomic analyses by providing more accurate identification of the genetic and functional potential of a microbial community.

## Introduction

Planetary protection (PP) requires a periodic assessment of the potential biological contamination for microbial burden of space flight hardware destined to or nearby planetary bodies that may or may not have harbored signs of life such as Mars or the Icy Worlds of the outer solar system (COSPAR, 2011). Space hardware exploring the possibility of life on a planetary body of interest, such as recent missions to Mars, are required to be cleaned to have less than an average of 300 spores/m^2^ on spacecraft surfaces and less than 5 × 10^5^ spores at launch (Benardini et al., 2014). For future missions, such as Mars Sample Return, in addition to limiting outbound contamination, the science community has deemed it important to collect an Earth-based assessment of the potential biological and organic contamination that the hardware could have experienced as part of the hardware integration and test operations (Beaty et al., 2018). Upcoming missions to the outer planets and the Icy Worlds, such as Europa Clipper, require less than 1 × 10^–4^ probability of contamination of the subsurface ocean by Earth-based microorganisms (National Research Council, 2000). Currently, the NASA standard assay (NSA) is used to test for spores on spacecraft, but this assay will not detect most microorganisms (NASA., 2010). Heat tolerant microorganisms (80°C; 15 min) that can grow aerobically in a nutrient rich Tryptic Soy Agar (TSA) medium, incubated at 32°C, for 72 h are captured by this NSA measurement, but slow growing microorganisms that (i) prefer cooler or hotter temperatures, (ii) are obligate anaerobes, (iii) or have dietary requirements not met by TSA may not be detected by this NSA method.

Metagenomics has been identified as an alternative biological verification technique to replace or supplement a culture-based assay that NASA could potentially use to detect the widest possible spectrum of microorganisms, including those of PP interest. NASA is seeking to develop this technology to demonstrate bioburden control assessments for enumeration of relevant viable organisms or contamination risk assessment. In doing so, this approach would likely include an enumeration, phylogenetic identification, and high-resolution characterization of microbial traits and biochemical capabilities from spacecraft surfaces. Microorganisms such as those which are radiation resistant, psychrophilic or anaerobic, etc., are likely to withstand space flight conditions (launch to landing) and may be able to survive on an extraterrestrial planet (forward contamination) (Thompson et al., 2017). High throughput metagenomics sequencing and bioinformatics pipelines have the potential to rapidly identify most of the microbial diversity (Horneck et al., 2012; Tamames and Puente-Sánchez, 2019) and to classify those microorganisms into PP relevant categories, but are not yet available.

The goal of this work is to test the ability of modern metagenomics pipelines to enable NASA in defining new PP requirements for detecting microorganisms on space hardware for future life detection missions. Proven modern molecular technologies are essential for NASA’s mission because none of the techniques used to clean and sterilize spacecraft components and subsystems (e.g., dry-heat sterilization, vapor hydrogen peroxide, oxygen plasma, etc.) are compatible with fully assembled, modern day spacecraft (Chung et al., 2008; Shirey et al., 2017). Hence, hitch-hiking microorganisms associated with spacecraft must be measured and characterized using modern molecular techniques throughout assembly of the spacecraft. Techniques like 16S rRNA gene cloning (1980s) (Pace, 1997), microarray (early 2000s) (Vaishampayan et al., 2010), and targeted amplicon sequencing (2010s) (Minich et al., 2018) were tried and utilized to measure the widest possible spectrum of microorganisms associated with spacecraft components, however, these molecular methods are unable to characterize functional pathways which are essential for identifying PP related microorganisms. Shotgun metagenomics sequencing and analyses pipelines can detect, quantify, and assess potential metabolisms of both cultivable and uncultivable microorganisms (Singh et al., 2018).

To test the ability of various metagenomics pipelines to detect the widest possible spectrum of microorganisms and of relevance to the PP mission, four vendors (labeled C, J, L, and N) were chosen to perform analyses of metagenomes generated from surface samples collected at the Jet Propulsion Laboratory (JPL) Spacecraft Assembly Facility (SAF) between March and August, 2016, just prior to the beginning of assembly of the Mars 2020 rover, a bioburden controlled and sample return biological contamination knowledge capture mission. These vendors are affiliated with a private industry (Vendor L), national laboratory (Vendor J), and academia (Vendor C, N), in the United States. The SAF is a good test environment for this project because (i) it is where Mars bound spacecraft have been assembled, (ii) it is a critical cleanroom for projects where component to assembly level integration and testing occurs, (iii) a rigorous cleaning regime and bio-control ensures that surface samples are low in biomass like samples from spacecraft surfaces, and (iv) it is a good testbed where microorganisms of PP concern could be detected and have been previously documented (Probst et al., 2010; Vaishampayan et al., 2012). Each vendor implemented their own metagenomics analysis pipeline and choose their own sequence databases for comparison without our interference, but all vendors were provided the same metagenomics sequence and associated metadata.

To measure the effectiveness of each pipeline, various requirements were considered. Quality control measures such as adapter removal, filtering of human reads, and sequence length and quality cutoffs were expected to be performed by each vendor. Control samples that displayed high or low similarity with other samples were included in the dataset sent to each vendor to test how controls were processed. Databases used by each vendor were considered for their ability to detect microorganisms of PP concern. The proportion of high-quality sequences assigned an annotation is also important, as a more comprehensive description of the sampled diversity clarifies the PP risk presented by detected microorganisms.

## Materials and Methods

### Description of Samples and Sample Selection Criteria

Among more than 200 samples collected from the JPL SAF clean room floors and control samples that were shotgun metagenome sequenced (Hendrickson et al., 2017), 20 were selected for this exercise (Table 1). These consist of handling and instrument controls, which include two dirty samples (FC2; 872,202 reads and MC2; 573,199 reads), and a clean sample (FC9; 5,471 reads). Additionally, SAF floor samples containing <1 × 10^6^ reads (*n* = 2; Group L) and >1 × 10^6^ reads (*n* = 15; Group H) were included. Among samples containing >1 × 10^6^ reads, three consist of microorganisms relevant for PP (Actinobacteria, Firmicutes, etc., Group H-F).

**TABLE 1:** Characteristics of samples analyzed.

						Vendor L			Vendor J			Vendor C			Vendor N	
Sample	Group	Location	Sample Type	Date of Sampling	Total number of raw reads	QC Reads^1^	Taxonomically Classified Reads^2^	>1%^3^	QC Reads^1^	Taxonomically Classified Reads^4^	>1%^3^	QC Reads^1^	Taxonomically Classified Reads^5^	>1%^3^	QC Reads^1^	Taxonomically Classified Reads^6^
FC2	Control	Field Control	Air of SAF	15-March-2016	872,202	74,023	338	332	320,075	9,263	9,163	41,880	9,911	9,705	15,726	N/A
FC9	Control	Field Control	Air of SAF	12-July-2016	5,471	232	–	–	704	44	9	401	44	44	83	N/A
MC2	Control	Maxwell Control	Extraction Control	15-March-2016	573,199	129,426	923	893	290,218	81,808	30,287	118,891	58,717	57,179	100,730	N/A
S14	L	L6	Floor of SAF	15-March-2016	529,438	108,702	225	222	259,750	31,984	2,669	101,610	4,406	3,813	77,363	N/A
S99	L	L11	Floor of SAF	26-July-2016	427,481	2,671	12,223 7	10,932	2,653	1,894	432	2,564	194	194	2,544	N/A
S12	H	L2	Floor of SAF	15-March-2016	1,097,438	787,877	1,108	1,061	869,207	267,667	48,750	564,322	59,647	45,850	676,490	N/A
S16	H	L5	Floor of SAF	15-March-2016	1,205,398	862,588	4,030	3,896	954,606	604,825	100,850	823,471	270,698	260,492	754,851	N/A
S17	H	L9	Floor of SAF	15-Mar-2016	10,647,036	9,726,824	26,421	25,298	9,782,262	1,176,911	108,555	9,733,552	502,672	458,315	8,680,384	N/A
S18	H	L13	Floor of SAF	15-March-2016	1,254,780	858,915	2,028	1,938	974,280	389,330	85,782	829,518	126,622	112,610	743,226	N/A
S20	H	L10	Floor of SAF	15-Mar-2016	2,001,909	1,584,542	4,593	4,371	1,644,129	1,001,993	240,780	1,535,396	283,615	205,900	1,305,244	N/A
S41	H	L1	Floor of SAF	17-May-2016	3,329,331	2,857,079	4,886	4,641	2,811,738	519,088	63,155	1,346,708	93,215	67,717	2,639,304	N/A
S42	H	L10	Floor of SAF	17-May-2016	2,726,783	2,212,223	3,793	3,497	2,208,614	803,127	68,856	1,261,498	204,248	153,587	2,056,000	N/A
S43	H	L8	Floor of SAF	17-May-2016	17,258,784	15,574,766	27,828	25,427	15,455,888	4,682,775	464,348	14,069,392	1,328,792	1,111,901	14,659,904	N/A
S44	H	L12	Floor of SAF	17-May-2016	1,335,957	1,104,044	1,310	1,291	1,099,278	312,955	46,684	729,636	56,630	43,348	1,025,038	N/A
S45	H	L10	Floor of SAF	17-May-2016	2,369,108	1,948,423	2,274	2,174	1,940,897	549,588	59,777	1,549,155	146,026	103,581	1,809,578	N/A
S57	H	L5	Floor of SAF	1-June-2016	2,661,331	2,319,103	8,192	7,743	2,317,306	1,205,865	238,697	2,166,598	218,332	147,781	2,189,283	N/A
S108	H	L9	Floor of SAF	16-August-2016	2,150,195	2,090,570	5,759	5,632	2,068,840	1,316,931	151,302	2,063,778	463,731	354,047	2,005,116	N/A
S29	H-F	L10	Floor of SAF	30-March-2016	4,117,535	3,477,442	21,199	19,808	3,568,720	2,324,729	316,881	3,229,576	1,996,816	1,926,851	3,050,205	N/A
S30	H-F	L1	Floor of SAF	30-March-2016	3,592,661	3,220,908	11,982	11,388	3,243,095	2,487,003	405,394	3,209,450	2,106,955	2,028,611	2,846,917	N/A
S76	H-F	L5	Floor of SAF	28-June-2016	14,027,039	12,697,322	5,797	5,418	12,636,003	1,332,390	136,865	12,709,423	251,337	211,846	12,079,811	N/A

Groups includes all control samples (*n* = 3), group [L]ow includes samples <1 × 10^6^ reads (*n* = 2), and group [H]igh includes samples >1 × 10^6^ reads (*n* = 15). A subset of group H, group H-F, includes samples known to be abundant in Actinobacteria and Firmicutes. ^1^Quality Control pipelines are detailed in section “Materials and Methods”. ^2^Proprietary pipeline of Vendor L. ^3^Low abundance taxa (<1% of a library) were removed from each vendor provided table to simplify comparison. ^4^Diamond BlastX/MEGAN6 analysis by Vendor J. ^5^KrakenUniq analysis by Vendor C. ^6^MetaPhlAn2 analysis by Vendor N provides relative abundance in terms of %, not absolute read counts. ^7^Anomaly.

Sample collection and processing methods were previously published (Hendrickson et al., 2017). Briefly, 1 m^2^ of floor was wiped with sterile, pre-moistened 23 × 23 cm polyester wipes (Texwipe; TX1009, Kernersville, NC, United States), and placed in a sterile bottle containing 200 mL phosphate buffer saline (PBS). The sample was mixed thoroughly, and the resulting particulates and microorganisms suspended in the PBS were concentrated with a CP-150 concentrating pipette (InnovaPrep, Drexel, MO, United States). DNA was extracted from concentrated samples using a Maxwell 16 (Promega, Madison, WI, United States) and sequenced using Illumina MiSeq (San Diego, CA, United States). Handling control samples (pre-moistened polyester wipes waved in the air of SAF) were also examined exactly like the SAF environmental samples. Reagent controls were also included during DNA extraction with the Maxwell 16 (well filled with PBS rather than concentrated sample).

### Metagenome Sequencing

Shotgun metagenome sequencing was carried out as described previously (Singh et al., 2018; Avila-Herrera et al., 2020). Briefly, DNA libraries were prepared for sequencing using the NextEra DNA Library Preparation Kit (Illumina, Inc., San Diego, CA, United States). Quality and fragment size were assessed on the Agilent Tapestation 4200 (Agilent Technologies, Santa Clara, CA, United States). Libraries were quantitated using the Qubit fluorimeter (Thermo Fisher Scientific, Waltham, MA, United States) and normalized to equivalent DNA quantities, pooled, and diluted according to the manufacturer’s standard recommendations. Shotgun metagenomic sequencing was performed using an Illumina NextSeq 500 with the NextSeq Series High Output Kit v2 (Illumina Inc., San Diego, CA, United States), using 150 base pair, paired end reads. Fastq files are generated from the sequencing results using the bcl2fastq software (Illumina) and given to vendors.

### Taxonomic and Functional Assignment

Each vendor was asked to perform their own taxonomic and functional assignment for all metagenomic reads provided to them using their preferred pipeline. Each vendor used different methods for quality control (QC) and classification of sequence data, used different databases for taxonomic and functional annotation. Detailed descriptions of the methodologies of each vendor are provided below.

### Vendor L Methods

#### Quality Control

To support multiple platforms, metadata included in the read files are analyzed to determine the appropriate QC pipeline and threshold settings. Settings are persisted to the database (for default values) and GUI support is implemented to allow users to enter custom threshold values for subsequent steps. An executable binary file is constructed and launched as either a parallel worker thread or parallel executor (Spark support) to construct an interactive HTML5 compliant page. This page is served to the user through workflow support in the GUI. Low quality reads are removed and cataloged (metadata to RDS) according to user-selected values.

#### Read Pairing

For sequencing technologies and protocols that support paired reads, similar sample paired read files are merged leveraging proprietary merge packages implemented in Java. Output is directed to a file system and file paths are persisted in the database allowing for simple GUI navigation.

#### Adapter Prediction

For paired reads that do not include known inserts and adapter sequences, the paired reads are run through a predictive algorithm. Metadata is pulled from the proprietary algorithm and persisted in the database.

#### Trimming

Merged paired reads are trimmed by either the known sequence input, or the predicted adapters that can be optionally generated. Trimmed, quality control screened files are then used in subsequent analysis using TrimGalore Version 0.6.5^1^.

#### Phylogenetic Analysis

High-throughput BLAST servers were set up on GPU-based AWS Elastic Cloud Compute (EC2) instances to support a high-throughput parallel read blast. Each individual read was mapped against libraries of known microbial genome assemblies. Reads that meet either default or user set thresholds can be aligned with all known microbial genomes and depending on similarity score can be assigned a taxonomic identification.

### Vendor J Methods

#### Metagenome Sequence Data Processing

Paired-end 150 bp reads were processed with Trimmomatic (Bolger et al., 2014) to trim adapter sequences and low-quality ends, with a minimum Phred score of 20 across the entire length of the read used as a quality cutoff. Reads shorter than 80 bp after trimming were discarded. All reads were normalized across samples as previously recommended (Nayfach and Pollard, 2016). High-quality filtered reads were clustered to respective taxonomic levels (domains through species) using the lowest common ancestor (LCA) algorithm provided by MEGAN6 (Huson et al., 2007).

#### Taxonomic and Functional Assignment

For lower downstream processing and visualization, the MEGAN6 metagenomics toolkit was used (Huson et al., 2017). The NCBI taxonomy database (Sayers et al., 2008), containing over 6.6 × 10^5^ reference sequences, and NCBI-NR protein sequence database, consisting of entries from GenPept, Swiss-Prot, PIR, PDB, and RefSeq, were used to assign taxonomic features to reads by using DIAMOND (Buchfink et al., 2015) and the weighted LCA algorithm of MEGAN6 (Huson et al., 2016). The identification of the reads to a taxon is not based on the genes only, but it is based on the comparison of the reads with the reference sequences deduced from the genomes of the curated NCBI taxonomy database (Sayers et al., 2008). Briefly, taxonomic and functional binning of the metagenomic reads is carried out using MEGAN6 (Huson et al., 2017), with the following settings: minScore = 50, maxExpected = 0.01, topPercent = 10, and minSupportPercent = 0.01. The resulting taxon assignments are presented in this manuscript. Functional analysis was carried out by mapping filtered DNA sequences against a reference database of all proteins within eggNOG (Powell et al., 2011), SEED (Overbeek et al., 2005), and Kyoto Encyclopedia of Genes and Genomes (KEGG) (Kanehisa and Goto, 2000) databases. The search for translated DNA sequences was executed using DIAMOND, and hits that spanned ≥20 amino acids with ≥90% similarity were retained. In cases where one read matched these criteria against multiple proteins, only the protein or proteins (in the event of a tie) with the maximum bit score were considered. Pathways were analyzed by summing counts of each KEGG orthology in a pathway. Using different databases allowed a detailed view of reads defined by gene function consisting of a collection of biologically defined (i) subsystems, (ii) clusters of orthologous groups, and (iii) collection of metabolic pathways.

### Vendor C Methods

#### Quality Control

Adapters and low-quality bases were removed using AdapterRemoval v2 (Schubert et al., 2016). Bases with a quality of 1 were removed as were ambiguous bases. Reads shorter than 50 bp after trimming were discarded. The remaining reads were aligned against the human genome with alternate contigs using Bowtie2 (Langmead and Salzberg, 2012) and “–sensitive” settings. Read pairs where only one read aligned were discarded.

Jellyfish (Marçais and Kingsford, 2011) was used to count k-mers in the processed reads. All k-mers, including singletons were counted. Various statistics were calculated on k-mers using a python script^2^. The two statistics presented here are (1) fraction of k-mers which are singletons, the number of k-mers which only occurred once vs. the total number of unique k-mers, and (2) k-mer entropy, Shannon entropy calculated over the probability of drawing each k-mer at random, as shown in previous work (Cleary et al., 2015; Costea et al., 2017).

Identification of likely negative controls from QC data was performed by manual inspection. Decision factors included the k-mer complexity (high singleton fraction, low entropy), the number of reads filtered, and taxonomic similarity to other samples (Levy and Borenstein, 2012; Thompson et al., 2017).

#### Taxonomic Profiling

Initial taxonomic profiling was performed by mapping clean-reads to all of RefSeq Microbial (1,977,559 unique taxa) using KrakenUniq (Breitwieser et al., 2018). KrakenUniq can produce false positive species calls, so we aggressively filtered results (McIntyre et al., 2017). We removed all species which were identified using fewer than 1,024 unique marker k-mers across the entire dataset. Additionally, species were removed if they were identified with fewer than 10,000 unique marker k-mers unless at least 10% of all known marker k-mers for that species were found. To provide an additional comparison, taxonomic profiles generated by MetaPhlAn2 (Segata et al., 2012) were also analyzed.

#### *De novo* Genome Assembly

Bacterial genomic sequences were assembled using metaSPAdes (Nurk et al., 2017), the best in-class metagenomic assembler. Resulting sequences were filtered for quality and duplicates, and annotated by aligning them to known sequences in the NCBI NT database. Annotations were processed using a series of GitHub scripts^3^ to identify a single likely annotation per outcome. Possible genes were annotated on the assembled *Acinetobacter* and *Bacillus* genomes (planetary protection relevant microbes), using PROKKA^4^. Many contigs, particularly larger contigs, did not precisely match any known taxa and may be from novel microbial strains.

#### Estimating Growth Rate

The estimated rate of growth for the two major taxa identified was evaluated using Growth Rate Index (GRiD) (Emiola and Oh, 2018). GRiD uses the peak to trough ratio of coverage on a microbial genome and a sophisticated series of filters to estimate that genomes rate of replication. GRiD is designed to work well even with low coverage samples and low-quality genome assemblies.

#### Functional Analysis

Microbial function was evaluated using the Human Microbiome Project (HMP) Unified Metabolic Analysis Network (HUMAnN2) (Franzosa et al., 2018). HUMAnN2 maps reads to UniRef90, a database of functional genes and combines genes into known metabolic pathways. Pathways are summarized by the total abundance of genes in the pathway and by the fraction of genes in the pathway which are identified. Pathways with less than 50% coverage were filtered from further analysis.

#### Comparison to MetaSUB Data

Vendor C used MetaSUB^5^, a large database of metagenomic samples from urban environments, to contextualize PP samples. This includes 2,126 MetaSUB samples with surface annotations and 371 MetaSUB samples collected from the air of six cities. These MetaSUB samples were processed for taxonomy and metabolic function analogously to how Vendor C processed the 20 samples in this study (*n* = 2,517 total).

After processing all 2,517 samples, taxonomic and functional profiles were reduced to a binary representation, indicating if a given taxa or pathway was detected in a given sample or not. Without performing this reduction of profiles to a binary representation, clean-room samples would cluster separate from MetaSUB samples. UMAP (McInnes et al., 2020) was used to perform dimensionality reductions for both taxonomy and function and observation of the results (Hart et al., 2015).

### Vendor N Methods

#### Sequencing Metrics and Quality Control

The sequencing quality of all 20 datasets was assessed using the publicly available FASTX-toolkit package^6^, as well as FastQC package^7^. Prior to analysis with MetaPhlAn2 and Kraken2 (see below), the paired reads were trimmed to removed adapters and low-quality bases using Trimmomatic (Bolger et al., 2014) with the following parameters: ILLUMINACLIP:/path/to/adapter.fasta:2:25:10 SLIDINGWINDOW:5:20 MINLEN:60.

#### Metagenomic Analysis of Sample Composition

All sequence reads were segmented into non-overlapping 50-mer fragments, which were then taxonomically classified down to the species level using the MTSv pipeline. The MTSv pipeline^8^ uses a local copy of NCBI’s GenBank database (accessed as of 06/15/2018) as its reference database. The pipeline performed a true alignment with up to three mismatches tolerated against all taxonomically classified sequences. Only the sequences that align to one-and-only-one taxonomic unit within the entirety of NCBI’s GenBank are used for taxonomic classification. In this fashion, only the sequence fragments that unambiguously support the presence of an organism are used, significantly reducing the false positive rate associated with metagenomic characterization of complex samples. Only organisms achieving at least 300 unique signature hits are reported.

The 20 metagenome datasets were also analyzed with MetaPhlAn2 (Segata et al., 2012), Kraken2 (Wood et al., 2019) and the downstream statistical module, Bracken (Lu et al., 2017). Bracken takes Kraken2 output and computes species abundance using Bayesian reestimation. In contrast to MetaPhlAn2, which relies upon a subset of markers for a given genome for classification, thereby limiting its ability to resolve low abundance organisms, Kraken2 is a k-mer based alignment against a reduced, but still large, version of RefSeq. Thus, Kraken2/Bracken is generally able to identify organisms in low abundance, but conversely is susceptible to false positives.

#### Functional Analysis of Samples

HUMAnN2 (Franzosa et al., 2018) is a tool for gene annotation metabolic pathway discovery from both metagenomic and metatranscriptomic data, and was developed by the same group that published MetaPhlAn2. HUMAnN2 generates three types of output: gene family abundance; pathway abundance; and pathway coverage. Each sample was run through HUMAnN2 where the analysis focused on gene family abundance for each genome that was discovered by MetaPhlAn2.

### Comparative Analyses of Vendor Results

All four vendors provided a table containing abundances for microbial species detected in each metagenomic libraries. Since some vendors provided species in addition to strain names while others provided only species names, all strain names were excised and abundances were summed as necessary to better facilitate a comparison between vendors. All read counts in the subsequent species tables were normalized by the number of annotated reads in each metagenomics library if not already performed by the vendor. Output tables from each vendor containing taxon abundance were merged together and utilized for comparative analyses.

Venn diagrams demonstrating overlap among species by the four vendors were generated using InteractiVenn (Heberle et al., 2015) for surface samples and controls separately. Analysis of similarities (ANOSIM) and non-metric multidimensional scaling (NMDS) comparing taxon tables from the four vendors were performed using the vegan R package (Oksanen et al., 2013) and custom R scripts^9^^,^^10^. Network diagrams for surface samples or controls were generated by averaging relative abundance for each taxon for each vendor and converting the resulting species matrix into a network using Cytoscape (v.3.8.0) (Shannon et al., 2003) and a custom Perl script^11^.

## Results

A key need for PP efforts is a rigorous genetic catalog of the presence of microorganisms on and around spacecraft associated environments, as well as an assessment of their likelihood of survival or persistence as well as proliferation in extraterrestrial conditions. To aid in this effort, we analyzed 20 PP-related samples for microbial taxonomy, metabolic function, growth rate, and assembled large genomic contigs. Shotgun metagenomics raw reads (BioProject PRJNA668809) were given to four different vendors to analyze bioinformatically using their favorite publicly available or in-house pipelines to hypothesize on the composition of the microbial community sampled. One of the main features of the analysis pipeline that deviated from other vendors was that Vendor J performed a protein-based taxonomic assignment, whereas other vendors carried out nucleotide-based taxonomic assignment. All vendors removed adapter sequences and human reads, retaining <1% (of control sample FC9) to >90% (of sample S108) of the total raw reads (7.22 × 10^7^; Table 1) which allowed for further taxonomic classification. To simplify comparative analyses, low abundance taxa identified by each vendor that comprised <1% relative abundance of a library were excised, reducing the effective number of reads analyzed by 0 to 92% (Table 1).

### Vendor L Results

**FIGURE 1:**
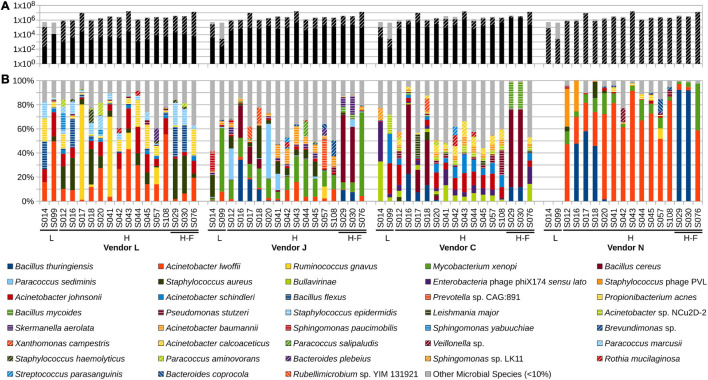
Abundance of reads in SAF floor samples that passed quality control (QC) measures of each vendor (black and black with cross-hatch; gray represents reads discarded by the QC stage; black without cross-hatch represent QC reads taxonomically classified) **(A)**, and relative abundance of species detected by each vendor **(B)**. Samples with a low- (group [L]ow; <1 × 10^6^) or high- (group [H]igh; >1 × 10^6^) numbers of reads were included. Samples with high numbers of reads associated with Actinobacteria and Firmicutes (group H-F) were also included. Vendor C used KrakenUniq and vendor N used MetaPhlAn2 for classifying the reads and assigning taxa.

**FIGURE 2:**
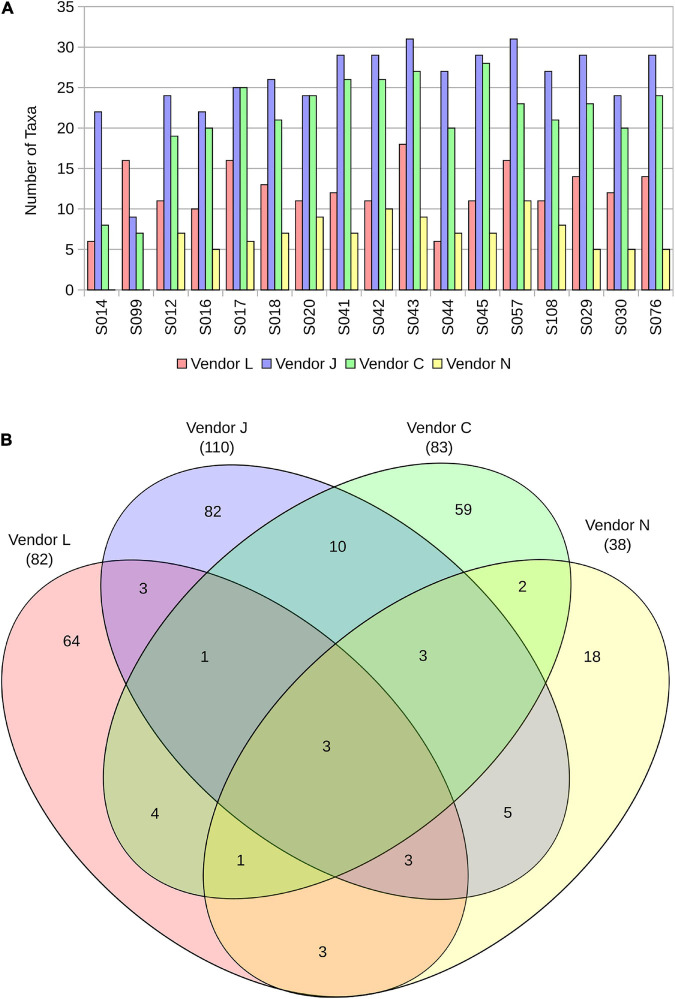
The number of taxa identified in each sample by the four vendors **(A)**, and a Venn diagram representing overlap in taxa detected in SAF floor samples **(B)**.

After QC steps followed by the Vendor L pipeline to remove adapter sequences and human reads generated 61,637,680 reads from 20 samples tested, and hence this vendor retained 85% of the raw reads for further analyses (Table 1, Figure 1, and Supplementary Figure 1A). The number of QC reads associated with 17 SAF floor samples ranged from 2,671 (S99) to 15,574,766 (S43), whereas the number of reads from field controls (*n* = 2) and Maxwell reagent control (*n* = 1) samples were less than 1,000. Only 0.22% of these QC reads were taxonomically classified by Vendor L. In general, Vendor L detected 82 species with high confidence (>1% relative abundance in at least one library) in the 17 SAF surface samples analyzed (Figure 2A), including 4 species of potential PP concern, *Bacillus flexus*, *Brevundimonas diminuta, Acinetobacter lwoffii*, and *A. johnsonii*. Additionally, Vendor L detected multiple species that are normally associated with the human skin and gut microbiome, including *Ruminococcus gnavus*, *Staphylococcus aureus*, *S. epidermidis*, and *Propionibacterium acnes*.

One of the field control samples (FC9) was found to possess only 232 reads after QC, hence no useful chromatogram could be generated to determine the distribution of species. In the other two control samples, Vendor L detected 11 species in Maxwell control (MC2) and 10 species in field control (FC2) samples (Supplementary Figure 1B). There were 2 bacterial species of potential PP concern [*Bacillus flexus* (83 reads) and *Anoxybacillus kestanbolensis* (61 reads)] found in Maxwell control (MC2) with very low number of reads. Similarly, Vendor L detected bacterial species associated with the human microbiome in controls samples (30–162 reads in FC2 and 28–489 reads in MC2), including *Staphylococcus aureus*, *S. epidermidis*, and *Propionibacterium acnes*.

During this study, Vendor L communicated that they have developed a proprietary pipeline to assemble genomes for unknown taxonomic units. Vendor L’s approach includes a method to determine contiguous regions of unmapped reads and to score and assess them for potential assembly into novel genomes of unknown species. However, such metagenome assembled genomes were not described by this vendor for this work. In addition, Vendor L failed to provide the results of their functional characterization.

### Vendor J Results

Among the total reads, ∼86% (62,448,263 reads) passed through the QC process (Fastp) of Vendor J (Table 1, Figure 1A, and Supplementary Figure 1A). After QC most of the reads were taxonomically classified as bacteria (85%), followed by eukaryotes (14%), then viruses (<1%), with few archaeal signatures detected (<0.1%). Vendor J detected 110 microbial taxa with high confidence (>1% relative abundance in at least one library) in the shotgun metagenome (Figure 1B). Dominating bacterial genera included *Bacillus*, *Acinetobacter*, *Bacteroides*, *Mycobacterium*, *Parabacteroides*, *Sphingomonas*, *Paracoccus*, *Lachnoclostridium*, *Clostridium*, and *Hungatella*. Vendor J paid special attention to detect Firmicutes and Actinobacteria, microorganisms that forms spores or are hardy; also, they are resistant to the extreme conditions (arid, radiation, etc.). *Bacillus*, *Lachnoclostridium*, and *Clostridium* were dominant Firmicutes, while *Mycobacterium*, *Corynebacterium*, and *Microbacterium* dominated *Actinobacteria*. Dominant bacteria detected are part of the human microbiome, which could be the reason for their abundance in the SAF environment.

Vendor J was able to taxonomically resolve to members of eukaryotes with >1% abundance in these samples, even though these cleanrooms were documented to be low in fungal diversity (La Duc et al., 2012). Among the total metagenomic reads of eukaryotes (14%), only half of them were associated with identifiable taxa having >1% relative abundance. Most of these eukaryotic sequences were Fungi, dominated by *Aureobasidium pullulans* and *Coniosporium apollinis*. Both the dominant fungi have been associated with low nutrient biomes (La Duc et al., 2012). Strains of *A. pullulans* have been isolated from SAF (La Duc et al., 2003), and *C. apollinis* is a rock inhibiting lithotroph (Sterflinger et al., 1997) that may be living in the cleanroom floor of the SAF. Similarly, less than 0.02% of archaeal and virus signatures were observed among the predominant metagenomic reads (>1%). Such low abundance of archaea and viruses in cleanrooms are well-known (Moissl et al., 2008).

Vendor J also inspected the genomic and metabolic capability of the microbial community members. To examine the presence of dormancy and sporulation genes, sequence reads from all samples were mapped to individual microbial genes, and then assigned to KEGG, SEED, and eggNOG categories. Genes associated with stationary phase, dormancy, and persistence were detected by Vendor J, including the HipAB system implicated in growth arrest, persistence, and drug tolerance, the Mycobacterial signal transduction system (MprAB) required for persistent infections, and a ribosomal hibernation related cluster. Additionally, multiple genes associated with sporulation were detected, including coat proteins (CotJABC), synthesis of dipicolinate and the exosporium, forespore to mother cell channel proteins, spore germination and germinant receptors, and other sporulation cluster genes like SigEG, SpoIIIAA-SpoIIIAH, SpoVA, and SpoVS (Supplementary Table 1). A low-biomass environment tends to have a majority of spore-forming populations (La Duc et al., 2007), and this environment is exhibiting presence of functional genes associated with spore-forming members. A descriptive result of eggNOG categories is presented in Supplementary Table 1D, mainly addressing the primary cellular function. KEGG analysis showed a much better input of the functional makeup of the microbial community. The presence of factors for “metabolism of terpenoids and polyketides,” “biosynthesis of other secondary metabolites,” “xenobiotics biodegradation and metabolism,” and “environmental adaptation,” represents a highly stressed and competitive environment. A closer look into the environmental adaption reveals the presence of the “bacterial secretion system” which is a strategy utilized by the pathogenic organism to infect the host. Our previous studies have shown that spacecraft associated environment is very similar to the nosocomial setup as both are maintained to near sterile levels using industrial cleaners (data not shown).

### Vendor C Results

Before any processing, the 20 samples contained a total of 72,183,076 reads. Of these, just over 10 million reads contained large Illumina adapter sequences. Vendor C removed adapters and low-quality bases using AdapterRemoval, thus reducing the total read count to 61,666,094 reads. Vendor C then removed human reads by aligning to the human reference genome using Bowtie2 (hg38, sensitive), and after removing reads where only one end aligned to the reference, Vendor C was left with 56,086,819 reads, 78% of the total (Table 1). Vendor C noted that this is a surprisingly small amount of human DNA for an environmental sample (Mason et al., 2016). Vendor C noted that FC9 and FC2 had noticeably lower complexity than other samples and correctly assumed they were negative controls. A third sample (MC2), was considered ambiguous by Vendor C due to its intermediate complexity. This MC2 sample was a Maxwell reagent control and Vendor C curation deemed correct in deciding which samples were control samples during this blind study.

Vendor C identified 82 relatively abundant taxa (>1%; Figure 1) in SAF floor samples, some of which are relevant for PP efforts. By abundance, these species principally came from 7 genera: *Bacillus*, *Acinetobacter*, *Paracoccus*, *Pseudomonas*, *Sphingomonas*, *Methylobacterium*, and *Brevundimonas*. Samples could be broadly clustered into three groups based on their taxonomic profile: the putative negative controls (two samples), *Acinetobacter*-dominated samples (eleven samples), and *Bacillus*-dominated samples (six samples). One sample, S14, was ambiguous. *Acinetobacter*-dominated samples tended to have higher taxonomic diversity than *Bacillus*-dominated samples. Dozens of these organisms are known to persist and survive in deserts, oceans, the arctic, or other harsh terrestrial environments, and are thus likely to be relevant for inter-planetary missions and planning. Vendor C identified a range of predicted phenotypes of PP interest, including: spore-forming, resistance to radiation, resistance to desiccation, halophilic, resistance to extreme pH, able to survive in cold temperatures (including cold waters), resistance to cleaning products, resistance to heavy metals, and an ability to metabolize unusual carbon sources.

Vendor C also used MetaSPAdes (Nurk et al., 2017) to generate assembled contigs from a variety of taxa. Long (>500 kbp) assembled contigs appear to be closely related to *Acinetobacter lwoffii* and *Bacillus cereus*, but do not precisely match any genome in the public databases. The long assembled contigs were covered by an average depth of 100X, indicating sufficient depth for accurate assembly. Contigs longer than 10 kbp were also assembled from a variety of other taxa. They noticed a large amount of *Pinus* sequences, but also assembled sequences from 20 plausible microbial taxa, including *Acinetobacter* and *Bacillus*. No sequence could be assembled from the two putative negative controls. GRiD was able to estimate the replication rate of the *Acinetobacter* assembly for all samples except controls (FC2, FC9, and MC2), and it was also able to estimate *Bacillus* replication rate for all samples with *Bacillus* (Supplementary Figure 2A). For both taxa, GRiD showed a high rate of replication (a GRiD score of 2 is a typical threshold for fast replication) in most samples. In samples where scores for both taxa could be obtained the GRiD scores for both taxa were similar, often with overlapping of the 95% confidence interval and similar mean estimates. Excluding two outliers, (S99 and S16) the mean estimate for each sample falls in a tight range. Techniques to estimate bacterial replication from metagenomic data are novel and have never been applied to low-complexity or clean-room data yet, and as such, thus these results should be viewed as preliminary.

The abundance of different functional pathways in samples was identified using HUMAnN2 (Franzosa et al., 2018) with UniRef90 (Suzek et al., 2014). Dimensionality reduction of the functional pathways showed a group of SAF samples clustering with MetaSUB air samples and a group of samples clustering with surface samples (Supplementary Figure 2B). However, these clusters did not correspond at the taxonomic level. Vendor C identified 48 biochemical and biological pathways that were differentially abundant in SAF samples (20 samples) compared to both MetaSUB-surface (2,126 samples) and MetaSUB-air samples (317 samples) (*p* < 0.01, Mann-Whitney-*U* test). These pathways were universally more abundant in SAF samples than MetaSUB samples, and may represent specific adaptation to the cleanroom environment. Enriched pathways were principally for nucleotide and protein biosynthesis, including *de novo* biosynthesis of adenosine, guanosine, L-valine, L-isoleucine, L-lysine, L-proline, L-arginine, and L-methionine. Additionally, pathways associated with metabolism which may be linked to new metabolic sources were also enriched, including glycolysis, pentose-phosphate-pathway, the glyoxylate bypass, acetylene degradation, and fermentation to isobutanol. In particular, nucleotide biosynthesis has been linked to bacterial resistance to radiation and desiccation (Cox and Battista, 2005).

### Vendor N Results

Primary quality control evaluation revealed that four of 20 samples (20%) were primarily composed of adapter dimer. One of these samples (S14) yielded no classifiable reads while the other three negative control samples (FC2, FC9, and MC2) showed considerably low number of taxa. Hence, Vendor N correctly concluded that FC2, FC9, and MC2 were negative controls, since the identity of the samples were not revealed to any vendors.

Taxonomy of the samples was determined using three methods: MTSv; MetaPhlAn2, and Bracken. MTSv uses an alignment method, which maps 50-mers from each sample to a local copy of the NCBI GenBank database. Metagenomic Phylogenetic Analysis (MetaPhlAn2) maps shotgun sequence reads to a database of clade-specific marker sequences and additionally determines relative abundance. The MetaPhlAn2 database contains one million genes that represent 17,000 reference genomes (bacterial, archaeal, viral, and eukaryotic). The third method, called Bayesian Reestimation of Abundance with Kraken (Bracken) performs k-mer mapping to a reduced version of the RefSeq database. All three of these methods provided generally similar results with limited but identifiable differences.

The MTSv method identified 189 taxa (155 bacteria, 4 fungi, 18 eukaryotes, and 2 viruses). Of these, 55 were called with very high confidence (40 bacteria, 14 eukaryotes, and 1 virus). MetaPhlAn2 called 121 bacteria, 1 eukaryote, and one virus. However, only 38 microbial taxa were found to be abundant (>1%; Figure 1) in SAF floor samples. Most of the bacterial genera and many of the species overlapped, but MTSv called species not identified by MetaPhlAn2 and the latter did not identify plant, *Homo sapiens* and other eukaryotes because they are not in the MetaPhlAn2 database. The Bracken results were more similar to those obtained using MetaPhlAn2, although additional species under the genera *Acinetobacter*, *Bacillus*, *Paracoccus*, *Pseudomonas*, and *Sphingomonas* were reported. A critical difference between read handling for MTSv and MetaPhlAn2 and Bracken is that MTSv reads were not trimmed prior to analysis, likely increasing overall sensitivity and decreasing specificity.

The relative abundance profiles provided by MetaPhlAn2 and Bracken revealed that in most cases, samples were composed of only a few dominant species and that those species were most frequently *Acinetobacter lwoffii*, a common skin inhabitant, or *Bacillus thuringiensis*, a common soil organism. Indeed, most of high abundance organisms identified by these methods were members of the human microbiome. In addition to these high abundance organisms, some low abundance organisms of interest were identified, such as the potentially radiotolerant *Methylobacterium radiotolerans* and *Deinococcus* sp., the UV resistant *Hymenobacter* sp. and the halotolerant *Kocuria rhizophila*. Several sphingomonads that have the potential to degrade hydrocarbons were also identified.

The bacteria identified were annotated using the KEGG and the HUMAnN2 tool. Vendor N examined the annotation tables for features associated with sporulation, peroxide resistance, halotolerance, cold shock, phosphate starvation and antibiotic and heavy metal resistance and discovered genes associated with all these functions (Supplementary Table 2). This includes 73 predicted sporulation proteins, including sigma factors, two component regulators, germination proteins, assembly proteins, stage-specific proteins, proteases, and coat proteins. A variety of heat shock proteins, including ECF sigma factors and chaperones, and cold shock proteins were annotated in numerous organisms. Catalase, heme and non-heme peroxidases, glutathione peroxidase and peroxiredoxin, which are all involved in resistance to peroxide were also found. Halotolerance factors, including glycine/betaine transporters or inositol monophosphatase, and genes associated with resistance to cadmium, camphor, chromate, cobalt, copper, fluoride, mercury, and tellurite were predicted. Additionally, genes associated with resistance to at least thirteen antibiotics, including vancomycin were found.

### Comparative Analyses of All Vendors

Analyses of taxonomic tables (Supplementary Table 3) produced by all four vendors (J, C, N, and L) reveals differences in classification methodologies used. Different QC metrics used by the four vendors resulted in different numbers of sequences analyzed in subsequent stages (Figure 1A and Supplementary Figure 1A). Analyses of species-level taxon tables using stacked bar plots of relative species abundance (Figure 1B and Supplementary Figure 1B) reveal the differences in taxonomic classification among vendors. The number of taxa identified in each sample by the four vendors are depicted in Figure 2A. Although there is overlap in species identified (Figure 2B), no two vendors report the same relative abundance (Figure 1), likely owing to different databases used during analysis. When using the same taxonomic classifier (MetaPhlAn2) with its own built-in database, there is a large similarity in relative abundance of species between vendor C and N that is easily visible in a stacked bar plot (Supplementary Figure 3), showing that earlier QC steps likely played a minor role in these different taxonomic classifications.

In low-biomass SAF floor samples (S014 and S099) that resulted in <1 × 10^6^ reads (sample group [L]ow), only vendors L, J, and C provided taxonomic classification. Vendor L describes these low-biomass samples as containing mainly human skin commensals, including *A. lwoffii* (16–50%), *A. johnsonii* (11–20%), *B. flexus* (1–23%), *P. acnes* (5–19%), *Phaeobacter gallaeciensis* (0–7%), *S. aureus* (0–4%), and *S. epidermidis* (1–14%). Vendor J describes these samples as containing *A. lwoffii* (<1–8%), *A. johnsonii* (<1-1%), *Cnuella takakiae* (0–9%), *S. aureus* (<1–18%), *Mycobacterium xenopi* (2–53%), *Sphingomonas paucimobilis* (0–15%), *Skermanella aerolata* (0–2%), and *A. baumannii* (1–3%). Vendor C describes these two low-biomass samples as containing *A. johnsonii* (1–25%), *A. schindleri* (<1–24%), *Acinetobacter* sp. NCu2D-2 (0–16%), *Acinetobacter* sp. TTH0-4 (0–7%), *Bullavirinae* (3–33%), *Enterobacteria* phage phiX174 *sensu lato* (3–33%), *Mycobacterium avium* (0–10%), and *Sphingomonas* sp. LK-11 (0–10%).

Taxonomic classifications for high-biomass samples collected from the SAF floor that resulted in >1 × 10^6^ reads (sample group H) were successfully provided by all four vendors without fail. Vendor L described these samples as being dominated by a diverse assemblage: *A. lwoffii* (1–55%), *A. johnsonii* (2–22%), *B. flexus* (0–24%), *Bacteroides fragilis* (0–5%), *B. plebeius* (0–10%), *Faecalibacterium prausnitzii* (0–8%), *Paracoccus aminovorans* (0–10%), *P. marcusii* (0–11%), *P. acnes* (<1–22%), *Rothia mucilaginosa* (0–4%), *Ruminococcus gnavus* (0–79%), *Sphingomonas yabuuchiae* (0–14%), *Staphylococcus aureus* (0–33%), *S. epidermidis* (0–19%), *S. haemolyticus* (0–11%), *Streptococcus infantis* (0–8%), and *Veillonella dispar* (0–7%). Vendor J also described these samples as being comprised of a diverse assemblage: an unidentified *Acidobacteria* sp. (0–6%), *A. baumannii* (<1–15%), *A. lwoffii* (<1–16%), *B. cereus* (<1–56%), *B. thuringiensis* (<1–34%), *Bacteroides plebeius* (0–5%), an unidentified *Brevundimonas* sp. (<1–13%), *Cellulomonas cellasea* (0–5%), *Comamonas terrigena* (0–6%), *Mycobacterium tuberculosis* (0–5%), *M. xenopi* (6–69%), *Paracoccus salipaludis* (<1–12%), *P. sediminis* (<1–45%), *Prevotella* sp. CAG:891 (0–24%), *Roseomonas nepalensis* (<1–10%), *Rubellimicrobium* sp. YIM 131921 (<1–10%), *Skermanella aerolata* (<1–15%), *S. aureus* (<1–27%), and *Xanthomonas campestris* (0–13%). Vendor C similarly described a diverse assemblage in the SAF floor samples, containing *A. baumannii* (<1–7%), *A. calcoaceticus* (<1–12%), *A. johnsonii* (<1–16%), *A. schindleri* (<1–15%), *Acinetobacter* sp. NCu2D-2 (<1–6%), *B. cereus* (0–64%), *B. mycoides* (0–22%), *B. thuringiensis* (0–23%), *Bullavirinae* (<1–14%), an unidentified *Dikarya* sp. (0–10%), *Enterobacteria* phage phiX174 *sensu lato* (<1–14%), *Leishmania major* (0–18%), *Mycobacterium avium* (<1–10%), *Negativicoccus massiliensis* (0–5%), *Pseudomonas stutzeri* (<1–21%), *Rothia mucilaginosa* (0–9%), *Streptococcus parasanguinis* (0–7%), and *Xanthomonas campestris* (0–10%). Vendor N described these samples as being composed of mainly *A. lwoffii* (3–91%), *B. thuringiensis* (<1–92%), *Bacteroides coprocola* (0–10%), *Mycobacterium xenopi* (2–39%), *Rickettsia felis* (0–9%), *Ruminococcus gnavus* (0–9%), *S. aureus* (0–13%), *Staphylococcus* phage PVL (0–32%), and an unidentified *Veillonella* sp. (0–12%).

High-biomass samples collected from the SAF floor that resulted in >1 × 10^6^ reads and contained a high abundance of Firmicutes or Actinobacteria (sample group H-F) were analyzed by all vendors. Vendor L described these samples as containing *A. johnsonii* (2–11%), *A. lwoffii* (2–19%), *B. flexus* (1–24%), *Faecalibacterium prausnitzii* (0–8%), *P. acnes* (<1–22%), *S. aureus* (4–33%), and *S. epidermidis* (1–19%). Vendor J described these samples as containing *B. cereus* (<1–56%), *B. thuringiensis* (<1–9%), *Mycobacterium xenopi* (6–69%), *Paracoccus sediminis* (<1–6%), and *Skermanella aerolata* (<1–15%) in abundance. Vendor C described these three samples as containing *B. cereus* (0–64%), *B. mycoides* (0–22%), *B. thuringiensis* (0–12%), *Bullavirinae* (<1–14%), an unidentified *Dikarya* sp. (0–10%), *Enterobacteria* phage phiX174 *sensu lato* (<1–14%), and *Mycobacterium avium* (<1–6%). Vendor N described these three samples as being dominated by *A. lwoffii* (3–59%), *B. thuringiensis* (0–92%), and *Mycobacterium xenopi* (2–39%).

A Venn diagram representing overlap in species detected in SAF floor samples by the four vendors (Figure 2B) demonstrates the similarity and differences between the methods used. All four vendors detected three microbial species in common from floor samples, including *A. johnsonii*, *P. stutzeri*, and *S. aureus*. Species detected by only three vendors include *A. lwoffii*, *B. thuringiensis*, *Bacteroides plebeius*, *E. coli*, *Haemophilus parainfluenzae*, *Rickettsia felis*, *Rothia mucilaginosa*, and *Ruminococcus gnavus*. All vendors classified species from floor samples not classified by other vendors, with Vendor L uniquely classifying 64 of 82 species (78%), Vendor J uniquely classifying 82 of 110 species (75%), Vendor C uniquely classifying 59 of 83 species (71%), and Vendor N uniquely classifying 18 of 38 species (47%).

**FIGURE 3:**
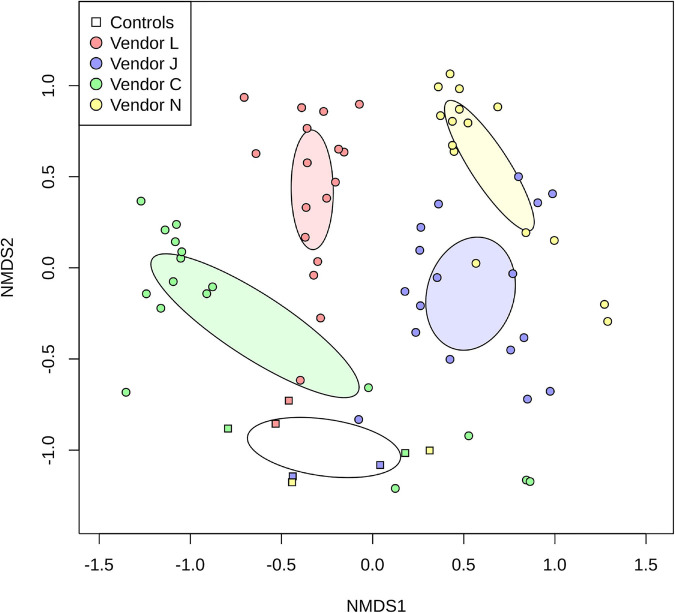
Non-metric multidimensional scaling (NMDS) plot representing differences in species detected in SAF floor samples (circle glyphs) and control samples (square glyphs). Lighter colored ellipses represent the 99% confidence interval for the mean of the SAF floor samples for each vendor. The empty ellipse represents the 99% confidence interval for the mean of the control samples for all vendors.

By plotting SAF floor and control samples with respect to the species classified by each vendor using non-metric multidimensional scaling (NMDS; Figure 3), the differences between methods used becomes very apparent. For the most part, point clusters representing SAF floor samples taxonomically classified by each vendor do not share the same ordination space with other vendors, with disjoint 99% confidence intervals for the sample mean (lightly colored ellipses). Control samples analyzed by all vendors (square glyphs) cluster separately (empty ellipse) from the SAF floor samples. Analysis of similarities of species classification among vendors shows that there is more similarity between samples analyzed by the same vendor than there is a similarity between vendors that is statistically significant (ANOSIM; *R* = 0.6056, *P* = 0.001).

All four vendors detected *S. aureus*, a skin-associated bacterium, as a dominant species in control samples (Supplementary Figure 1B). *S. aureus* makes up 34–97% of handling control sample FC2 (4.2 × 10^4^ reads), and 14–66% of Maxwell reagent control MC2 (1.56 × 10^5^ reads). *B. thuringiensis, B. cereus*, and *B. flexus* which are closely related, spore-forming, soil-associated bacteria, also made up a large proportion (9–66%) of Maxwell reagent control sample MC2. However, Vendor L only identified *B. flexus* in this sample and Vendor N only identified *B. thuringiensis*, while Vendor J and Vendor C identified closely related *B. cereus* and *B. thuringiensis*.

Analysis of overlap in the dominant species (>1%) detected in control samples (kitome) by each vendor (Supplementary Figure 1C) reveals limited similarity. However, the number of taxonomically classified reads (>1%) in these libraries were extremely low, ranging from 9 to 57,179 reads. All four vendors detected one species in common, *S. aureus*. Vendor C, Vendor N, and Vendor J additionally detected *B. thuringiensis*. All vendors but Vendor N uniquely identified species in the control samples: Vendor L uniquely identified 13 of 17 species, Vendor J uniquely identified 41 of 50 species, and Vendor C uniquely identified 12 of 19 species.

### Comparative Analyses of All Vendors on Planetary Protection Relevant Microbial Species

The Space Studies Board suggested classifying microbial species based on the level of concern that the species could survive unintentional transport via spacecraft and colonize a planetary body such as Mars or Jupiter’s icy moon Europa (National Research Council, 2000). To this end, they created four categories: Type A, microorganisms cultivable on the NSA without heat shock; Type B, spore-forming microorganisms (NSA heat-shock); Type C, radiation-resistant (10% survival at 0.8 Mrad), spore-forming microorganisms; and Type D, radiation-resistant (10% survival at 4.0 Mrad), non-spore-forming microorganisms. Although microorganisms classified as Type C and Type D are the most likely to survive space flight, we consider microorganisms classified as Type B, Type C, and Type D, or extremophiles suited to mission-specific environments as potentially PP relevant microbial species for this analysis.

**FIGURE 4:**
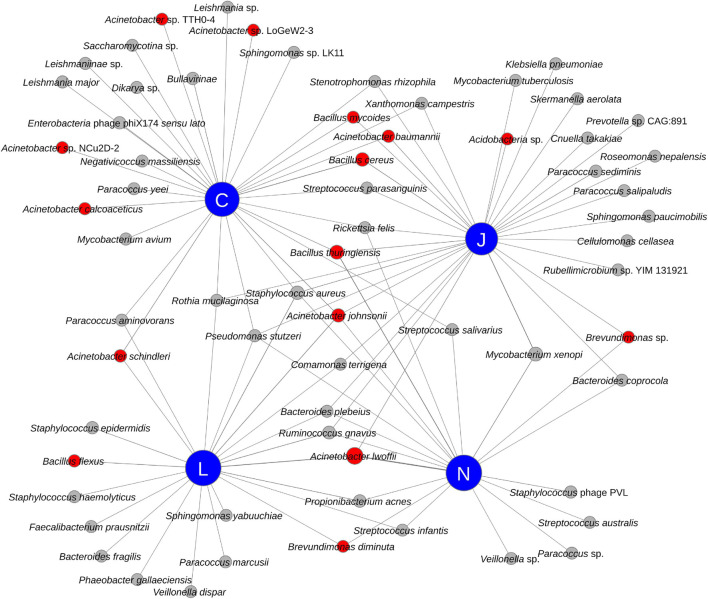
Network diagram showing the relationship between vendors (blue nodes) and detected species (red and gray nodes). Species of potential Planetary Protection concern have red colored nodes while others have gray colored nodes.

All four vendors detected bacteria that may be relevant for planetary protection purposes (see red nodes in Figure 4). Radiation resistant bacteria associated with *Acinetobacter* (*A. baumannii*, *A. johnsonii*, *A. lwoffii*, and *A. schindleri*), *Brevundimonas diminuta*, and an unidentified *Brevundimonas* sp. were detected by multiple vendors in SAF samples. Additionally, Vendor C detected other *Acinetobacter* species in SAF floor samples, including *A. calcoaceticus* and other *Acinetobacter* species (strains LoGeW2-3, NCu2D-2, and TTH0-4). An unidentified *Acidobacteria* sp., which could be an extremophile (Kielak et al., 2016), was detected by Vendor J in SAF floor samples. Notably, all four vendors detected members of *Bacillus*, a genus of microorganisms of PP concern with spore-forming capabilities. *B. cereus* was detected by Vendor J and Vendor C; *B. flexus* was detected by Vendor L; *B. mycoides* was detected by Vendor J and Vendor C; and *B. thuringiensis* was detected by Vendors C, N, and J.

Among the functional characteristics, genes associated with dormancy and sporulation pathways were detected by Vendors J and N. These include sigma factors, two component regulators, germination proteins, assembly proteins, stage-specific proteins, proteases, and coat proteins. Additionally, these two vendors detected gene pathways involved in resistance to antimicrobial agents, heavy metals, and resistance to stress (e.g., aerotolerance, heat shock, cold shock, and osmotic stress), potentially showing adaptation of these microorganisms to the clean-room environment.

In field control sample (FC2), spore-forming bacteria including *B. cereus*, *B. flexus*, and *B. thuringiensis* were also detected but in low numbers. In addition, the spore-forming *Anoxybacillus kestanbolensis* was also detected in the Maxwell reagent control sample (MC2) by Vendor L.

## Discussion

NASA PP efforts are concerned with hitch-hiking microorganisms that may have the capability to withstand spaceflight conditions and contaminate a planetary body of scientific interest. Concerns regarding the inability of the NSA culture method to detect all microorganisms in a sample has led NASA to look toward state-of-the art molecular methods like shotgun metagenomics to detect a wider range of taxa on spacecraft and associated environments. All four vendors used in this study identified taxa of PP concern in the JPL SAF environment where spacecraft are built, including *Acidobacteria*, *Acinetobacter, Anoxybacillus*, *Bacillus, Brevundimonas*, and *Caulobacteraceae*, demonstrating that modern metagenomic approaches and computational biology can detect these organisms of concern, unlike NSA culture methods.

Although similar species were detected by the four vendors, one issue noted by these analyses is that the relative abundances varied drastically between vendors. Different QC measures likely played a minor role in this since Vendor C and N used different QC methods but arrived at similar results when using the same taxonomic classifier (MetaPhlAn2; Supplementary Figure 3). The largest contributors to this difference in relative abundance is likely due to the different programs and databases used. Although Vendor L, Vendor J, and Vendor C used similar sequence-based analyses, their results varied drastically due to different databases used. Vendor C and Vendor N both used MetaPhlAn2 with its built-in database to arrive at very similar results, but different from the results produced by Vendor J and Vendor L, and Vendor C when using KrakenUniq. MetaPhlAn2 reports relative abundances in terms of the number of cells (Segata et al., 2012) rather than fraction of reads as done by the other analyses, likely contributing to this observed difference.

By ignoring relative abundance calculations and simply examining the overlap in species detected (Figure 2B), the difference between vendors becomes clearer. The overlap in species identified in SAF floor samples between the four vendors is small (three species). Vendor J and Vendor C shared the largest overlap in species identified (17 species). Vendor J identified more species than other vendors (110), likely due to the vast size of the database they used. All vendors uniquely identified species not identified by the other vendors. Failure to detect microorganisms present in a sample is concerning for PP purposes, especially if the missed microorganisms could survive on a spacecraft and go on to contaminate a planetary body of scientific interest. Utilization of large databases containing a wide breadth of available reference genome sequences may help to alleviate this issue.

Falsely detected species may also be of concern, especially if the detected species is of potential PP concern. Falsely detecting such a species would needlessly require extra rounds of cleaning, negatively affect the schedule of a spacecraft build, and potentially delay its launch. The large number of uniquely detected species by each vendor is especially concerning in this regard. Some of these uniquely identified species may have simply been miscategorized due to lack of a better alternative in the databases used or sequence fragments aligning to genomic regions with poor phylogenetic resolution. Adding a requirement for minimum coverage of a genome prior to classifying a species as present in a sample may help to limit such false positives. In addition, computational analysis at the genus level may also solve this problem since short reads of metagenome sequences may not have the taxonomic resolution to differentiate taxa at the species level.

Some of the same species seen in SAF floor samples can be seen in control samples, alluding to potential contamination of sampling equipment (e.g., the wipes) or reagents. Simply excising species with an abundant number of reads found in control samples from environmental samples is an option, but this may be problematic for PP purposes given that some of the species detected in the control samples are organisms of potential PP concern. The QC read counts were extremely low in these control samples, and the PP relevant microorganisms detected (*Bacillus* spp. and *Anoxybacillus kestanbolensis*) likely had a minor influence on the species detected in the floor samples. Compared to control samples, QC reads from floor samples were much more abundant (Table 1). Among control samples, *S. aureus* constituted 47–97% of a sample, whereas *S. aureus* constituted 1–11% in corresponding floor samples. Furthermore, viability assessment of the microbes would help to eliminate the kitomes from the calculations. In addition to pretreating with propidium monoazide (Vaishampayan et al., 2013), application of novel technique such as GRiD analysis would eliminate naked DNA sequences associated with kitomes. In this study, Vendor C was able to estimate the replication rate of some taxa from SAF samples but such analysis did not show replication of taxa in control samples (FC2, FC9, and MC2).

The variance in relative abundance seen between vendors may make metagenomic assays problematic for PP purposes without additional work on calibration of these metagenomic pipelines. The ability to estimate the number of problematic microorganisms on a spacecraft surface is currently hampered by these large variations between methods and databases used, but is paramount for determining the amount of bioburden on these surfaces. Synthetic metagenomes containing sequence fragments in known quantities have been developed (McIntyre et al., 2017), and can be utilized for this purpose. However, it would be beneficial to develop synthetic metagenomes from the genomes of microorganisms that PP efforts are concerned with, in addition to microorganisms previously detected in PP-relevant samples, to better test and calibrate these pipelines. Since SAF cleanrooms are known to be similar to other built environments with regular human movement (Lax et al., 2014), inclusion of microorganisms known to inhabit these environments in a synthetic metagenome would be important. Additionally, procedural changes that include spiking a known quantity of DNA into samples prior to sequencing should be considered to allow for more precise estimates of microbial abundance (Hardwick et al., 2018; Blackburn et al., 2019).

Only ∼15% of the QC sequences were utilized for identification of species by all vendors except Vendor L (<1%), likely due to the non-availability of annotated genomes in public databases. Public databases are mainly comprised of cultivable microorganisms with annotations, whereas very few genomes of non-cultivable microorganisms are present. To enable the utilization of discarded shotgun metagenome reads, metagenome assembled genomes (MAG) of microorganisms present in NASA cleanroom samples should be generated, allowing for annotation of their genetic capabilities and detection of PP-relevant metabolisms. Since spacecraft associated surfaces are low in biomass (Minich et al., 2018), MAGs should additionally be generated from the samples collected from PP relevant extreme environments such as radionucleotide dumping sites, nuclear accidents, and hot and cold arid regions of the Earth. Additionally, MAGs should be generated from samples collected from built-environments that do not undergo strict cleaning regimes and are directly adjacent to the relevant cleanrooms, to ensure that the genetic repertoire of microorganisms that may hitch-hike between the two are captured. MAGs from such environments would help to identify and annotate these uncultivable microorganisms to decipher the metabolic and functional pathways and expand the understanding of species diversity of spacecraft and associated environments.

This study was performed using short metagenomic sequences (∼150-bp) generated using Illumina technology, which is known to function with low-biomass clean room samples. The vast number of sequences that can be produced by this technology usually offsets its main drawback, the short length of the sequences. Short sequences are often harder to identify with certainty because they align to highly conserved segments of a genome and provide no specific information, or because they represent novelty that is not yet represented in a public database. Longer sequences generated by technologies developed by Pacific Biosciences (PacBio) and Oxford Nanopore Technology (ONT) provide an opportunity to more accurately identify a larger portion of the sequences generated because the ambiguous segments are often linked to segments that can be identified. Technologies that generate longer sequences should be considered for future metagenomic studies of spacecraft hardware to aid in identification of PP relevant taxa. The issues associated with long read technologies like ONT MinION or PacBio are that these methodologies require higher concentration of DNA as well as longer fragment length. Such issues can be mitigated when larger surface areas are collected and gentle sample processing technologies developed to get longer fragment DNA length. These tasks are not trivial to perform in samples collected from an extremely oligotrophic, clean, low-biomass spacecraft and associated environments.

Each vendor in this study utilized different computational algorithms and pipelines to analyze the provided shotgun metagenome data, and here we attempt to highlight some of the benefits and downfalls of each approach. Vendor C and Vendor N used multiple methods for taxonomic identification (Vendor C used KrakenUniq and MetaPhlAn2; Vendor N used MTSv, MetaPhlAn2, and Kraken/Braken), which allowed them to have more confidence in species identified via multiple methods. However, Vendor N did not provide taxon tables for their MTSv or Kraken/Braken results, so comparative analyses with these pipelines were not possible. The taxon table provided by Vendor L identified <1% of QC sequences, with an anomaly in sample S99 where 12,223 reads were taxonomically classified, but only 2,671 reads passed their QC stage (458% identified), bringing into question the overall quality of their results. Vendor J used a massive database (NCBI-NR) that allowed them to identify species not detected by other vendors, however, the degenerate nature of protein sequences (with to regard to DNA sequences) means that their pipeline would have a hard time differentiating between two closely related microorganisms. Vendors L, C, and N all used databases containing DNA sequences for identification potentially allowing for more specific results, but increased computation time to analyze DNA sequences limited the size of databases that could be used. The custom database used by Vendor L to analyze the shotgun metagenome sequences was too limited in genetic breadth to accurately describe the diversity present in the samples provided to them. Vendor C and Vendor J generated MAGs from the dataset provided, giving these vendors more confidence in the species identified. These MAGs additionally allowed Vendor C to estimate the rate of growth of these microorganisms by using the GRiD software package to map that ratio of DNA close to the origin of replication with that of the terminus region. Although no results were provided, Vendor L claims to be working on novel machine-learning/artificial-intelligence algorithms that could help increase accuracy of results.

## Conclusion

Although metagenomics offers tantalizing access to the genetic and functional potential of a microbial community, more work needs to be carried out to standardize ever evolving analyses pipelines and databases used to understand big-data generated from a low-biomass environment. To standardize these metagenomics sequence analyses pipelines, synthetic metagenomes containing known quantities should be developed and utilized. Additionally, the use of sequencing technologies that produce longer sequences should be considered to aid in the identification of PP-relevant microorganisms. Even though all the vendors in this study utilized different computational algorithms and pipelines to analyze the provided shotgun metagenome data, Vendor J approaches allowed them to identify species not detected by other vendors. Invariably, all vendors utilized could only resolve about 15% of the shotgun sequences since public databases contain only annotated sequences pertaining to culturable microorganisms. Metagenomics has the potential to be a useful assay for detecting microorganisms of PP concern and explore functional characteristics of yet-to-be cultured microorganisms by constructing MAGs.

## Data Availability Statement

Publicly available datasets were analyzed in this study. This data can be found here: BioProject PRJNA668809.

## Author Contributions

KV and JB coordinated with all authors in designing the concept, executed the study, implemented the project, involved in data analyses, communicated with all vendors in producing the data, and involved in the logistical efforts of the work. JW and KV wrote the manuscript. JW generated figures and tables for the manuscript and performed all statistical analyses. NS implemented some of the bioinformatics analyses. AS generated a figure for the manuscript. LG and AS curated metadata and organized the sequence data. All authors contributed to the article and approved the submitted version.

## Conflict of Interest

The authors declare that the research was conducted in the absence of any commercial or financial relationships that could be construed as a potential conflict of interest.

## Publisher’s Note

All claims expressed in this article are solely those of the authors and do not necessarily represent those of their affiliated organizations, or those of the publisher, the editors and the reviewers. Any product that may be evaluated in this article, or claim that may be made by its manufacturer, is not guaranteed or endorsed by the publisher.

## Footnotes

1
https://github.com/FelixKrueger/TrimGalore

2
https://github.com/dcdanko/gimmebio/blob/master/gimmebio/kmers/gimmebio/kmers/cli.py

3
https://github.com/dcdanko/gimmebio/blob/develop/gimmebio/assembly/gimmebio/assembly/cli.py

4
https://github.com/tseemann/prokka

5
http://www.metasub.org

6
http://hannonlab.cshl.edu/fastx_toolkit/

7
https://www.bioinformatics.babraham.ac.uk/projects/fastqc/

8
https://github.com/FofanovLab/MTSv

9
https://github.com/sandain/R/blob/main/anosim.R

10
https://github.com/sandain/R/blob/main/mds.R

11
https://github.com/sandain/pigeon/blob/main/scripts/species-matrix2graphml.pl

## Supplementary Material

Supplementary Figure 1 Abundance of reads in control samples that passed quality control (QC) measures of each vendor (dark- and light-blue; gray represents reads discarded by the QC stage; dark-blue represent QC reads taxonomically classified) **(A)**, relative abundance of species detected by each vendor **(B)**, Venn diagram representing overlap in species detected in control samples **(C)**, and network diagram showing the relationship between vendors (blue nodes) and detected species (red and gray nodes; red nodes are species of potential Planetary Protection concern) in control samples **(D)**. The FC9 sample is not included due to the low number of reads (*n* = 44) as well as their taxonomic affiliation to the genus and species was not resolved.

Supplementary Figure 2 Estimated replication score for assembled *Acinetobacter* and *Bacillus* species in each sample **(A)**. Higher scores indicate faster replication, range indicates the 95% confidence interval. Some samples were not scored for one or both species because that species was not detected. Values are similar when reference genomes are substituted for assemblies. Red glyphs are *Acinetobacter* and blue are *Bacillus*. Comparison of cleanroom samples with the MetaSUB database **(B)**. Both panels (left and right) show cleanroom samples plotted with MetaSUB samples as the background. Plotting was performed using UMAP to reduce data to two dimensions. The input data were binary, indicating presence or absence of a feature. Left shows microbial species, right shows microbial metabolic pathways.

Supplementary Figure 3 Relative abundance of species detected by Vendor C and Vendor N when both vendors use the same taxonomic classification algorithm (MetaPhlAn2).

Supplementary Table 1 Dormancy and sporulation subsystems **(A)** and all subsystems **(B)** detected by vendor J using the SEED database.

Supplementary Table 2 Genes and predicted proteins associated with sporulation, heat shock, peroxide resistance, halotolerance, cold shock, phosphate starvation, and antibiotic and heavy metal resistance detected by vendor N using HUMAnN2.

Supplementary Table 3 Taxonomic classification of SAF floor samples **(A)** and control samples **(B)** performed by each vendor.

## References

[B1] Avila-Herrera A. Thissen J. Urbaniak C. Be N. A. Smith D. J. Karouia F. (2020). Crewmember microbiome may influence microbial composition of ISS habitable surfaces. *PLoS One* 15:e0231838. 10.1371/journal.pone.0231838 32348348 PMC7190111

[B2] Beaty D. W. Mcsween H. Y. Carrier B. L. Czaja A. D. Goreva Y. S. Hausrath E. M. (2018). “Analysis of the Scientific Value of the Mars2020 Spacecraft Genetic Inventory to Mars Sample Return,” in *49th Lunar and Planetary Science Conference*, (Houston, TX).

[B3] Benardini J. N. La Duc M. T. Beaudet R. A. Koukol R. (2014). Implementing planetary protection measures on the mars science laboratory. *Astrobiology* 14 27–32. 10.1089/ast.2013.0989 24432776

[B4] Blackburn J. Wong T. Madala B. S. Barker C. Hardwick S. A. Reis A. L. M. (2019). Use of Synthetic DNA spike-in controls (sequins) for human genome sequencing. *Nat. Protoc.* 14 2119–2151. 10.1038/s41596-019-0175-1 31217595

[B5] Bolger A. M. Lohse M. Usadel B. (2014). Trimmomatic: a flexible trimmer for Illumina sequence data. *Bioinformatics* 30 2114–2120. 10.1093/bioinformatics/btu170 24695404 PMC4103590

[B6] Breitwieser F. P. Baker D. N. Salzberg S. L. (2018). KrakenUniq: confident and fast metagenomics classification using unique K-mer Counts. *Genome Biol.* 19:198. 10.1186/s13059-018-1568-0 PMC6238331 30445993

[B7] Buchfink B. Xie C. Huson D. H. (2015). Fast and sensitive protein alignment using DIAMOND. *Nat. Methods* 12 59–60. 10.1038/nmeth.3176 25402007

[B8] Chung S. Kern R. Koukol R. Barengoltz J. Cash H. (2008). Vapor hydrogen peroxide as alternative to dry heat microbial reduction. *Adv. Space Res.* 42 1150–1160. 10.1016/j.asr.2008.01.005

[B9] Cleary B. Brito I. L. Huang K. Gevers D. Shea T. Young S. (2015). Detection of low-abundance bacterial strains in metagenomic datasets by eigengenome partitioning. *Nat Biotechnol.* 33 1053–1060. 10.1038/nbt.3329 26368049 PMC4720164

[B10] COSPAR (2011). *COSPAR Planetary Protection Policy.* Houston, TX: World Space Council.

[B11] Costea P. I. Zeller G. Sunagawa S. Pelletier E. Alberti A. Levenez F. (2017). Towards standards for human fecal sample processing in metagenomic studies. *Nat. Biotechnol.* 35 1069–1076. 28967887 10.1038/nbt.3960

[B12] Cox M. M. Battista J. R. (2005). *Deinococcus radiodurans* — the consummate survivor. *Nat. Rev. Microbiol.* 3 882–892. 10.1038/nrmicro1264 16261171

[B13] Emiola A. Oh J. (2018). High throughput *in situ* metagenomic measurement of bbacterial replication at ultra-low sequencing coverage. *Nat. Commun.* 9:4956. 10.1038/s41467-018-07240-8 PMC6251912 30470746

[B14] Franzosa E. A. Mciver L. J. Rahnavard G. Thompson L. R. Schirmer M. Weingart G. (2018). Species-level functional profiling of metagenomes and metatranscriptomes. *Nat. Methods* 15 962–968. 10.1038/s41592-018-0176-y 30377376 PMC6235447

[B15] Hardwick S. A. Chen W. Y. Wong T. Kanakamedala B. S. Deveson I. W. Ongley S. E. (2018). Synthetic microbe communities provide internal reference standards for metagenome sequencing and analysis. *Nat. Commun.* 9:3096. 10.1038/s41467-018-05555-0 PMC6078961 30082706

[B16] Hart Y. Sheftel H. Hausser J. Szekely P. Ben-Moshe N. B. Korem Y. (2015). Inferring biological tasks using pareto analysis of high-dimensional data. *Nat. Methods* 12 233–235. 10.1038/nmeth.3254 25622107

[B17] Heberle H. Meirelles G. V. Da Silva F. R. Telles G. P. Minghim R. (2015). InteractiVenn: a web-based tool for the analysis of sets through venn diagrams. *BMC Bioinform.* 16:169. 10.1186/s12859-015-0611-3 25994840 PMC4455604

[B18] Hendrickson R. Lundgren P. Malli Mohan G. B. Urbaniak C. Benardini J. N. Venkateswaran K. (2017). “Comprehensive Measurement of Microbial Burden in Nutrient-Deprived Cleanrooms,” in *47th International Conference on Environmental Systems*, (Charleston, SC: International Conference on Environmental Systems).

[B19] Horneck G. Moeller R. Cadet J. Douki T. Mancinelli R. L. Nicholson W. L. (2012). Resistance of bacterial endospores to outer space for planetary protection purposes—experiment PROTECT of the EXPOSE-E Mission. *Astrobiology* 12 445–456. 10.1089/ast.2011.0737 22680691 PMC3371261

[B20] Huson D. H. Auch A. F. Qi J. Schuster S. C. (2007). MEGAN analysis of metagenomic data. *Genome Res.* 17 377–386. 10.1101/gr.5969107 17255551 PMC1800929

[B21] Huson D. H. Beier S. Flade I. Górska A. El-Hadidi M. Mitra S. (2016). MEGAN community edition - interactive exploration and analysis of large-scale microbiome sequencing data. *PLoS Comput. Biol.* 12:e1004957. 10.1371/journal.pcbi.1004957 27327495 PMC4915700

[B22] Huson D. H. Tappu R. Bazinet A. L. Xie C. Cummings M. P. Nieselt K. (2017). Fast and simple protein-alignment-guided assembly of orthologous gene families from microbiome sequencing reads. *Microbiome* 5:11. 10.1186/s40168-017-0233-2 PMC5267372 28122610

[B23] Kanehisa M. Goto S. (2000). KEGG: kyoto encyclopedia of genes and genomes. *Nucleic Acids Res.* 28 27–30. 10592173 10.1093/nar/28.1.27 PMC102409

[B24] Kielak A. M. Barreto C. C. Kowalchuk G. A. Van Veen J. A. Kuramae E. E. (2016). The ecology of acidobacteria: moving beyond genes and genomes. *Front. Microbiol.* 7:744. 10.3389/fmicb.2016.00744 27303369 PMC4885859

[B25] La Duc M. T. Dekas A. Osman S. Moissl C. Newcombe D. Venkateswaran K. (2007). Isolation and characterization of bacteria capable of tolerating the extreme conditions of clean room environments. *Appl. Environ. Microbiol.* 73 2600–2611. 10.1128/aem.03007-06 17308177 PMC1855582

[B26] La Duc M. T. Nicholson W. Kern R. Venkateswaran K. (2003). Microbial characterization of the mars odyssey spacecraft and its encapsulation facility. *Environ. Microbiol.* 5 977–985. 10.1046/j.1462-2920.2003.00496.x 14510851

[B27] La Duc M. T. Vaishampayan P. Nilsson H. R. Torok T. Venkateswaran K. (2012). Pyrosequencing-derived bacterial, archaeal, and fungal diversity of spacecraft hardware destined for mars. *Appl. Environ. Microbiol.* 78 5912–5922. 10.1128/aem.01435-12 22729532 PMC3406123

[B28] Langmead B. Salzberg S. L. (2012). Fast gapped-read alignment with Bowtie 2. *Nat. Methods* 9 357–359. 10.1038/nmeth.1923 22388286 PMC3322381

[B29] Lax S. Smith D. P. Hampton-Marcell J. Owens S. M. Handley K. M. Scott N. M. (2014). Longitudinal analysis of microbial interaction between humans and the indoor environment. *Science* 345 1048–1052. 10.1126/science.1254529 25170151 PMC4337996

[B30] Levy R. Borenstein E. (2012). “Reverse Ecology: From Systems to Environments and Back,” in *Evolutionary Systems Biology*, ed. Soyer O. (New York, NY: Springer). 10.1007/978-1-4614-3567-9_15 22821465

[B31] Lu J. Breitwieser F. P. Thielen P. Salzberg S. L. (2017). Bracken: estimating species abundance in metagenomics data. *PeerJ Comput. Sci.* 3:e104. 10.7717/peerj-cs.104 PMC12016282 40271438

[B32] Marçais G. Kingsford C. (2011). A fast, lock-free approach for efficient parallel counting of occurrences of K-mers. *Bioinformatics* 27 764–770. 10.1093/bioinformatics/btr011 21217122 PMC3051319

[B33] Mason C. Afshinnekoo E. Ahsannudin S. Ghedin E. Read T. Fraser C. (2016). The Metagenomics and metadesign of the subways and urban biomes (MetaSUB) international consortium inaugural meeting report. *Microbiome* 4:24. 10.1186/s40168-016-0168-z PMC4894504 27255532

[B34] McInnes L. Healy J. Melville J. (2020). *UMAP: uniform manifold approximation and projection for dimension reduction.* Ithaca, NY: arXiv e print.

[B35] McIntyre A. B. R. Ounit R. Afshinnekoo E. Prill R. J. Hénaff E. Alexander N. (2017). Comprehensive benchmarking and ensemble approaches for metagenomic classifiers. *Genome Biol.* 18:182. 10.1186/s13059-017-1299-7 PMC5609029 28934964

[B36] Minich J. J. Zhu Q. Janssen S. Hendrickson R. Amir A. Vetter R. (2018). KatharoSeq enables high-throughput microbiome analysis from low-biomass samples. *mSystems* 3 e217–e218. 10.1128/mSystems.00218-17 PMC5864415 29577086

[B37] Moissl C. Bruckner J. C. Venkateswaran K. (2008). Archaeal diversity analysis of spacecraft assembly clean rooms. *ISME J.* 2 115–119. 10.1038/ismej.2007.98 18180750

[B38] NASA. (2010). *Handbook for the Microbiological Examination of Space Hardware, NASA-HDBK-6022.* Washington, DC: National Aeronautics and Space Administration.

[B39] National Research Council. (2000). *Preventing the Forward Contamination of Europa.* Washington, DC: The National Academies Press.

[B40] Nayfach S. Pollard K. S. (2016). Toward accurate and quantitative comparative metagenomics. *Cell* 166 1103–1116. 10.1016/j.cell.2016.08.007 27565341 PMC5080976

[B41] Nurk S. Meleshko D. Korobeynikov A. Pevzner P. A. (2017). MetaSPAdes: a new versatile metagenomic assembler. *Genome Res.* 27 824–834. 10.1101/gr.213959.116 28298430 PMC5411777

[B42] Oksanen J. Blanchet F. G. Kindt R. Legendre P. Minchin P. R. O’hara R. B. (2013). *Vegan: Community Ecology Package”. 2.0-10 ed.* Available online at: https://CRAN.R-project.org/package=vegan (accessed Nov 28, 2020).

[B43] Overbeek R. Begley T. Butler R. M. Choudhuri J. V. Chuang H. Y. Cohoon M. (2005). The subsystems approach to genome annotation and its use in the project to annotate 1000 Genomes. *Nucleic Acids Res.* 33 5691–5702. 10.1093/nar/gki866 16214803 PMC1251668

[B44] Pace N. R. (1997). A molecular view of microbial diversity and the biosphere. *Science* 276 734–740. 10.1126/science.276.5313.734 9115194

[B45] Powell S. Szklarczyk D. Trachana K. Roth A. Kuhn M. Muller J. (2011). eggNOG v3.0: orthologous groups covering 1133 organisms at 41 different taxonomic ranges. *Nucleic Acids Res.* 40 D284–D289. 22096231 10.1093/nar/gkr1060 PMC3245133

[B46] Probst A. Vaishampayan P. Osman S. Moissl-Eichinger C. Andersen G. L. Venkateswaran K. (2010). Diversity of anaerobic microbes in spacecraft assembly clean rooms. *Appl. Environ. Microbiol.* 76 2837–2845. 10.1128/aem.02167-09 20228115 PMC2863428

[B47] Sayers E. W. Barrett T. Benson D. A. Bryant S. H. Canese K. Chetvernin V. (2008). Database resources of the national center for biotechnology information. *Nucleic Acids Res.* 37 D5–D15. 18940862 10.1093/nar/gkn741 PMC2686545

[B48] Schubert M. Lindgreen S. Orlando L. (2016). AdapterRemoval v2: rapid adapter trimming, identification, and read merging. *BMC Res. Notes* 9:88. 10.1186/s13104-016-1900-2 26868221 PMC4751634

[B49] Segata N. Waldron L. Ballarini A. Narasimhan V. Jousson O. Huttenhower C. (2012). Metagenomic microbial community profiling using unique clade-specific marker genes. *Nat. Methods* 9 811–814. 10.1038/nmeth.2066 22688413 PMC3443552

[B50] Shannon P. Markiel A. Ozier O. Baliga N. S. Wang J. T. Ramage D. (2003). Cytoscape: a software environment for integrated models of biomolecular interaction networks. *Genome Res.* 13 2498–2504. 10.1101/gr.1239303 14597658 PMC403769

[B51] Shirey T. B. Schubert W. Benardini J. (2017). “An Overview of Surface Heat Microbial Reduction as a Viable Microbial Reduction Modality for Spacecraft Surfaces,” in *47th International Conference on Environmental Systems*, (Charleston, S C: International Conference on Environmental System).

[B52] Singh N. K. Wood J. M. Karouia F. Venkateswaran K. (2018). Succession and persistence of microbial communities and antimicrobial resistance genes associated with International Space Station environmental surfaces. *Microbiome* 6:214. 10.1186/s40168-018-0609-y PMC6280456 30514368

[B53] Sterflinger K. De Baere R. De Hoog G. S. De Wachter R. Krumbein W. E. Haase G. (1997). *Coniosporium perforans* and *C. apollinis*, two new rock-inhabiting fungi isolated from marble in the Sanctuary of Delos (Cyclades. Greece). *Antonie van Leeuwenhoek* 72 349–363. 9442275 10.1023/a:1000570429688

[B54] Suzek B. E. Wang Y. Huang H. Mcgarvey P. B. Wu C. H. The Uniprot Consortium. (2014). UniRef Clusters: a comprehensive and scalable alternative for improving sequence similarity searches. *Bioinformatics* 31 926–932. 10.1093/bioinformatics/btu739 25398609 PMC4375400

[B55] Tamames J. Puente-Sánchez F. (2019). SqueezeMeta, a highly portable, fully automatic metagenomic analysis pipeline. *Front. Microbiol*:9:3349. 10.3389/fmicb.2018.03349 30733714 PMC6353838

[B56] Thompson L. R. Sanders J. G. Mcdonald D. Amir A. Ladau J. Locey K. J. (2017). A communal catalogue reveals earth’s multiscale microbial diversity. *Nature* 551 457–463. 29088705 10.1038/nature24621 PMC6192678

[B57] Vaishampayan P. Osman S. Andersen G. Venkateswaran K. (2010). High-Density 16S microarray and clone library–based microbial community composition of the phoenix spacecraft assembly clean room. *Astrobiology* 10 499–508. 10.1089/ast.2009.0443 20624058

[B58] Vaishampayan P. Probst A. J. La Duc M. T. Bargoma E. Benardini J. N. Andersen G. L. (2013). New perspectives on viable microbial communities in low-biomass cleanroom environments. *ISME J.* 7 312–324. 23051695 10.1038/ismej.2012.114 PMC3554398

[B59] Vaishampayan P. A. Rabbow E. Horneck G. Venkateswaran K. J. (2012). Survival of *Bacillus pumilus* spores for a prolonged period of time in real space conditions. *Astrobiology* 12 487–497. 10.1089/ast.2011.0738 22680694

[B60] Wood D. E. Lu J. Langmead B. (2019). Improved metagenomic analysis with kraken 2. *Genome Biol.* 20:257. 10.1186/s13059-019-1891-0 PMC6883579 31779668

