# Supplementary figures and images for: Performance of Multiple Metagenomics Pipelines in Understanding Microbial Diversity of a Low-Biomass Spacecraft Assembly Facility
Source: Front Microbiol. 2021 Sep 28;12:685254. doi: 10.3389/fmicb.2021.685254 (PMC8508200; doi:10.3389/fmicb.2021.685254)

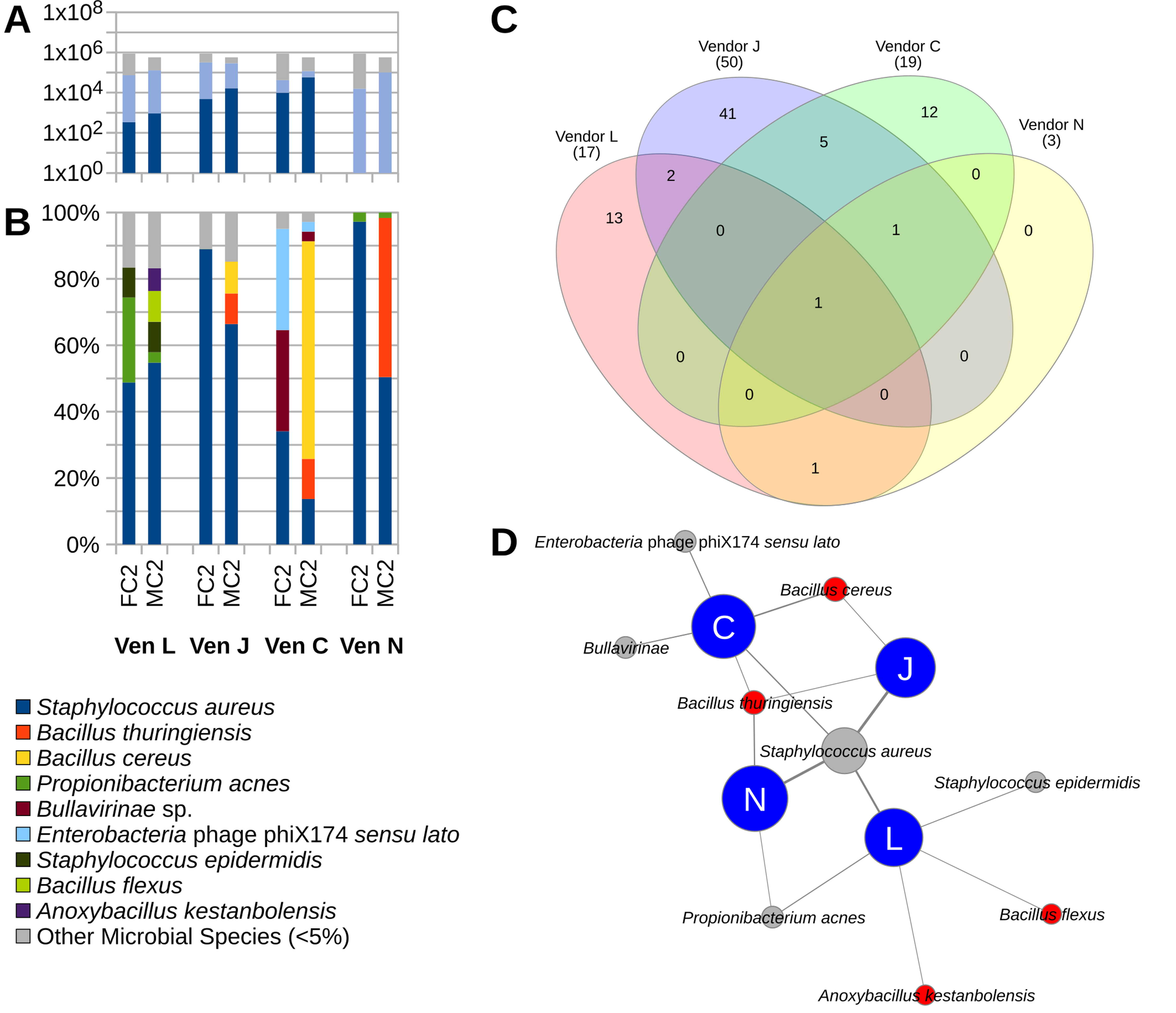

Supplement: Supplementary Figure 1 — Abundance of reads in control samples that passed quality control (QC) measures of each vendor (dark- and light-blue; gray represents reads discarded by the QC stage; dark-blue represent QC reads taxonomically classified) (A), relative abundance of species detected by each vendor (B), Venn diagram representing overlap in species detected in control samples (C), and network diagram showing the relationship between vendors (blue nodes) and detected species (red and gray nodes; red nodes are species of potential Planetary Protection concern) in control samples (D). The FC9 sample is not included due to the low number of reads (n = 44) as well as their taxonomic affiliation to the genus and species was not resolved. [file Image_1.TIFF]

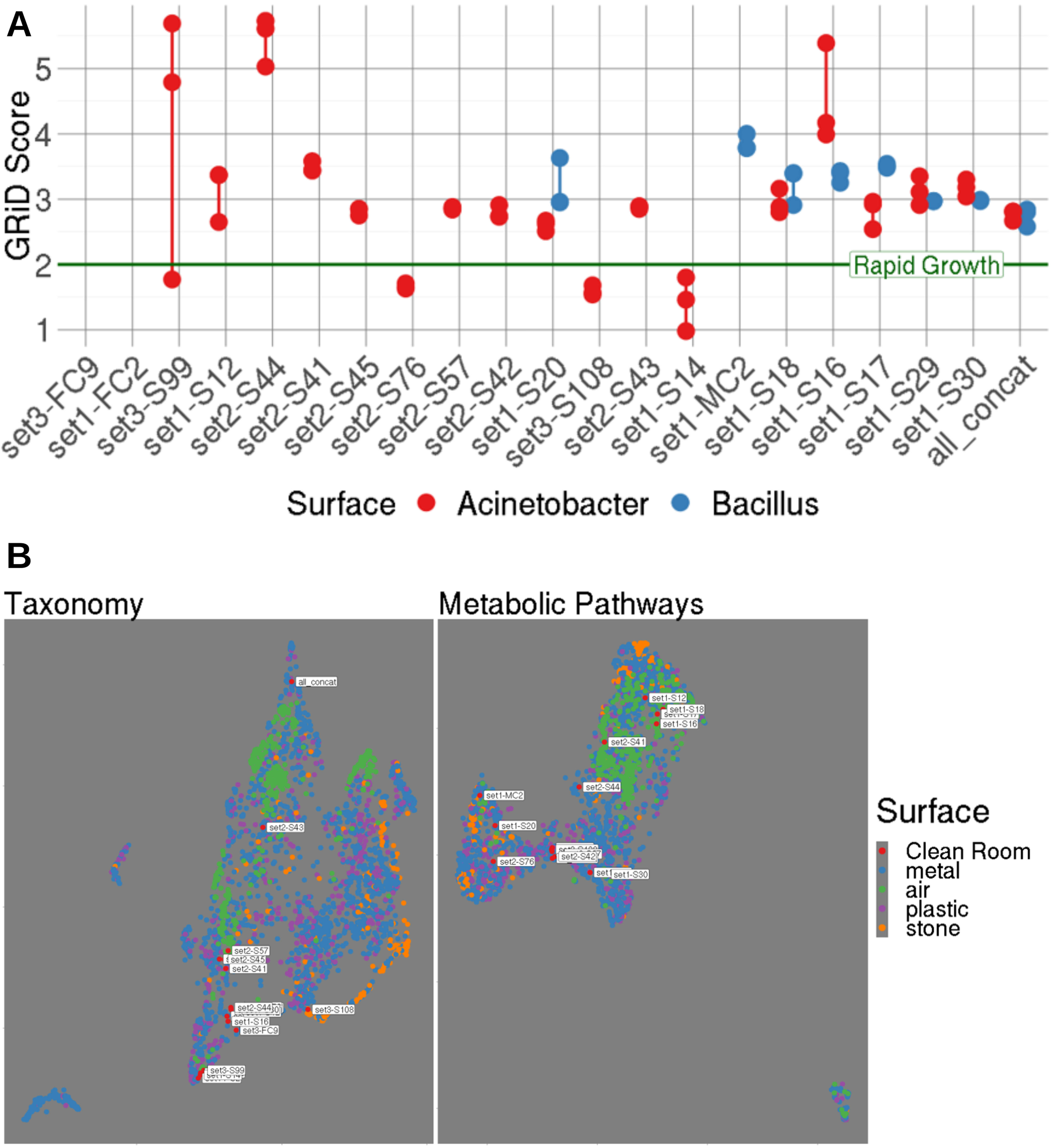

Supplement: Supplementary Figure 2 — Estimated replication score for assembled Acinetobacter and Bacillus species in each sample (A). Higher scores indicate faster replication, range indicates the 95% confidence interval. Some samples were not scored for one or both species because that species was not detected. Values are similar when reference genomes are substituted for assemblies. Red glyphs are Acinetobacter and blue are Bacillus. Comparison of cleanroom samples with the MetaSUB database (B). Both panels (left and right) show cleanroom samples plotted with MetaSUB samples as the background. Plotting was performed using UMAP to reduce data to two dimensions. The input data were binary, indicating presence or absence of a feature. Left shows microbial species, right shows microbial metabolic pathways. [file Image_2.TIFF]

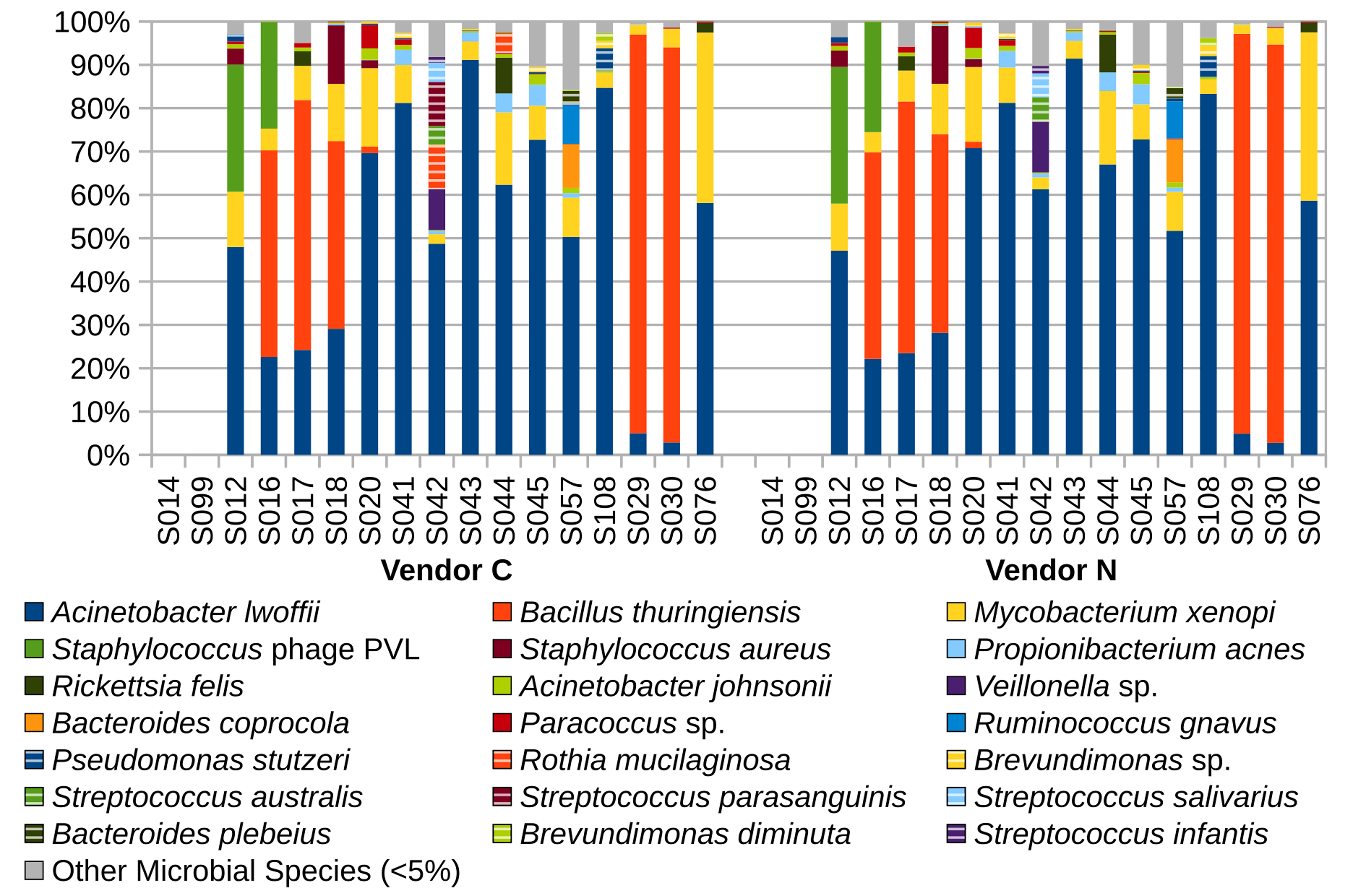

Supplement: Supplementary Figure 3 — Relative abundance of species detected by Vendor C and Vendor N when both vendors use the same taxonomic classification algorithm (MetaPhlAn2). [file Image_3.TIFF]
